# Mechanism of Blood–Heart-Barrier Leakage: Implications for COVID-19 Induced Cardiovascular Injury

**DOI:** 10.3390/ijms222413546

**Published:** 2021-12-17

**Authors:** Rubens P. Homme, Akash K. George, Mahavir Singh, Irina Smolenkova, Yuting Zheng, Sathnur Pushpakumar, Suresh C. Tyagi

**Affiliations:** Department of Physiology, University of Louisville School of Medicine, Louisville, KY 40202, USA; rubens.petithomme@louisville.edu (R.P.H.); akash.kunnumelkudygeorge@louisville.edu (A.K.G.); irina.smolenkova@louisville.edu (I.S.); yuting.zheng@louisville.edu (Y.Z.); sbpush01@louisville.edu (S.P.); suresh.tyagi@louisville.edu (S.C.T.)

**Keywords:** congestive heart failure, creatinine kinase isoforms, COVID-19 viral myocarditis, matrix metalloproteinases, tissue remodeling

## Abstract

Although blood–heart-barrier (BHB) leakage is the hallmark of congestive (cardio-pulmonary) heart failure (CHF), the primary cause of death in elderly, and during viral myocarditis resulting from the novel coronavirus variants such as the severe acute respiratory syndrome novel corona virus 2 (SARS-CoV-2) known as COVID-19, the mechanism is unclear. The goal of this project is to determine the mechanism of the BHB in CHF. Endocardial endothelium (EE) is the BHB against leakage of blood from endocardium to the interstitium; however, this BHB is broken during CHF. Previous studies from our laboratory, and others have shown a robust activation of matrix metalloproteinase-9 (MMP-9) during CHF. MMP-9 degrades the connexins leading to EE dysfunction. We demonstrated juxtacrine coupling of EE with myocyte and mitochondria (Mito) but how it works still remains at large. To test whether activation of MMP-9 causes EE barrier dysfunction, we hypothesized that if that were the case then treatment with hydroxychloroquine (HCQ) could, in fact, inhibit MMP-9, and thus preserve the EE barrier/juxtacrine signaling, and synchronous endothelial-myocyte coupling. To determine this, CHF was created by aorta-vena cava fistula (AVF) employing the mouse as a model system. The sham, and AVF mice were treated with HCQ. Cardiac hypertrophy, tissue remodeling-induced mitochondrial-myocyte, and endothelial-myocyte contractions were measured. Microvascular leakage was measured using FITC-albumin conjugate. The cardiac function was measured by echocardiography (Echo). Results suggest that MMP-9 activation, endocardial endothelial leakage, endothelial-myocyte (E-M) uncoupling, dyssynchronous mitochondrial fusion-fission (Mfn2/Drp1 ratio), and mito-myocyte uncoupling in the AVF heart failure were found to be rampant; however, treatment with HCQ successfully mitigated some of the deleterious cardiac alterations during CHF. The findings have direct relevance to the gamut of cardiac manifestations, and the resultant phenotypes arising from the ongoing complications of COVID-19 in human subjects.

## 1. Introduction

Numerous studies have focused on CHF, but a very few have determined the BHB function [1]. The endothelium, whether it is in the endocardium or in a coronary or capillary, is the primary barrier against BHB dysfunction [2,3,4]. Regarding the tight-junction proteins such as connexin-37 between endothelial-endothelial, connexin-43 between endothelial-myocyte, myocyte-myocyte and mitochondria (mito)-myocyte are the primary connexins [5,6,7]. In this context, researchers have started investigating that it is important to determine the mechanism of BHB leakage if the same are involved in various disease conditions including the viral infections, including the COVID-19 that infect vital organs such heart [1,8,9,10,11,12,13,14]. It is true that remodeling by its very nature implies synthesis and degradation of extracellular matrix (ECM). Hence, matrix metalloproteinases (MMPs) are known to play significant role in vascular leakage and interstitial edema [15,16,17,18,19]. The basement membrane between endothelium and muscle contains ECM, encompassing the latent MMPs and tissue inhibitor of metalloproteinases (TIMPs)/nitric oxide; NO- as the ternary complex. However, oxidative stress during CHF activates MMPs and inactivates TIMPs via peroxynitrite and tyrosine/arginine nitosylation [20]. The disruption of juxtacrine endothelial-myocyte (E-M), myocyte-myocyte (M-M) and mitochondria (mito)-myocyte uncoupling are the hallmarks of cardiac failure [3,21,22,23,24,25,26]. The proposed study investigated the role of connexin-43 which connects myocyte-myocyte and mitochondria (mito)-myocyte in a mouse model of CHF that was created by AVF [27,28,29,30,31]. The connexin-37 connects the endothelial-myocyte. In E-M, M-M and mito-myocyte uncoupling, the role of MMPs in degradation of connexins causing BHB dysfunction remains unclear.

Although well-done, randomized clinical trials are needed urgently to test potential therapies including HCQ once the pharmacokinetics and pharmacodynamics (PK/PD) of HCQ are fully understood for the identification of an effective and safe therapeutic regimen for treatment of patients, we decided to study the potential mechanism(s) in an animal model, in anticipation of any benefits, if at all, regarding repurposing of HCQ in treating viral infections such as COVID-19 [32,33,34,35,36]. It is known that chloroquine (CQ) inhibits MMPs activity, endothelial hyperpermeability and by doing so it stops the leakage [37,38,39,40,41,42,43,44,45,46,47,48,49,50,51,52,53]. The mechanism(s) of action of CQ or its derivatives are still being worked out because they are not yet fully elucidated [54,55,56]. MMPs play crucial roles in tissue damage that occurs during remodeling. While studying patients with systemic lupus erythematosus (SLE) in patients that were sex- and age-matched with healthy volunteers’ serum levels of MMP-9 and TIMP-1 were determined, the same procedure was performed after CQ treatment. It was found that serum levels of MMP-9 were higher in patients compared with the controls. After CQ therapy the median MMP-9 serum levels in SLE patients decreased significantly suggesting that CQ treatment may affect the MMP-network and that this effect may contribute to immunoregulatory and anti-inflammatory properties of the antimalarials [45]. CQ also has anti-invasive effects since it suppresses the MMP-2 and MMP-9 mRNA expression levels and the protein activity, but the expression and proteolytic activity of MMP-13 mRNA usually increases as revealed in mice that were given CQ intraperitoneally [46]. Further, HCQ was able to protect mice against infection induced lethality by reducing cytokine production, endothelial hyperpermeability and the vascular leakage [47,57].

We know that CQ is a weak base and that is trapped upon protonation in the acidic compartments such as late endosomes and lysosomes, wherein it alkalizes these compartments thus affecting the fusion events and disrupting the endosomal and autophagic cargo degradation. In this way, it blocks lysosomal function. By rendering these functions, CQ normalizes vessel structure, their function and helps increase the perfusion. CQ’s vessel normalization activity relies on alterations of endosomal Notch1 trafficking and signaling in the endothelial cells [49,50,51,52]. Our study will test the concept that complications in viral myocarditis induced CHF can be mitigated by HCQ; a more potent derivative of CQ. Currently, several drugs are being tried to relive the symptoms of COVID-19 including dapagliflozin (SGLT2-antidiabetic) lopinavir/ritonavir, darunavir/umifenovir (anti-HIV), remdesivir (anti-Ebola), favipiravir and dipyridamole (anti-hypertensive) [41,58,59,60,61,62,63]. Additionally, an anti-acid (famotidine) is being tested [64,65,66,67]. Interestingly, HCQ is unique in the sense that it mitigates viral illnesses; therefore, we will employ the low dose HCQ to mitigate the CHF and viral myocarditis-induced illness. A clinical study compared suppressive effects of dipyridamole and CQ on SARS-CoV-2replication and suggested similar titer beginning at 100 nM [58]. We will use this concentration in vivo. Since cardiac matrix is unique, thus a cardio specific MMP inhibitor may mitigate the BHB leakage and dilated cardiomyopathy (DCM) [1]. Therefore, a cardiac specific MMP inhibitor (i.e., IMP) is needed to reduce mortality related to COVID-19 [68,69,70,71]. The role of HCQ in cardiac and skeletal muscle remodeling is novel since the mitigation of systemic remodeling during CHF by HCQ appears to be an innovative approach. In this context, the cardiac specific MMP-9 that can be inhibited by HCQ, is therapeutically novel too. To determine endothelial-dependent and independent cardiac function, the cardiac ring preparation was developed and pioneered in our lab is novel again [72]. We opine that HCQ treatment will be like the treatment with doxycycline D, a suggested MMP inhibitor for remodeling since it reverses the EE dysfunction [73,74]. The hypothesis is that activation of MMP-9 causes EE barrier dysfunction and the treatment with HCQ inhibits MMP-9 and preserves EE barrier/juxtacrine, and synchronous endothelial-myocyte coupling as also recently seen in the COVID-19 patients wherein high levels of MMPs including the MMP-9 are associated with the disease severity [75,76,77,78,79].

## 2. Results

### 2.1. Effects of HCQ on Heart and Lung Weights

**Figure 1:**
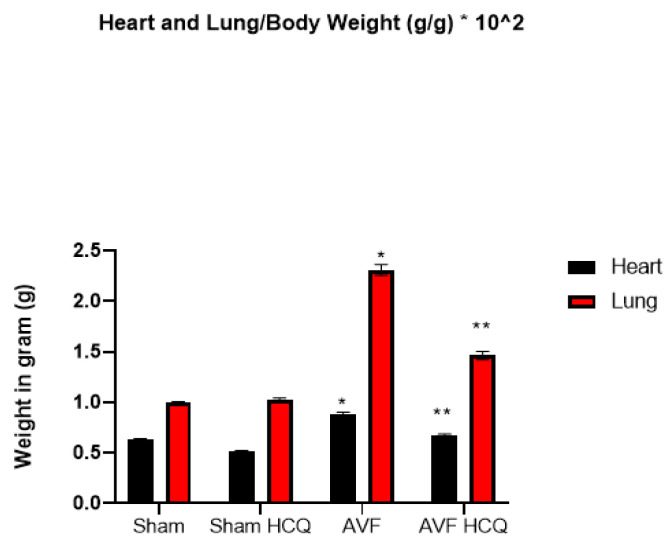
Heart and lung weights of AVF and sham mice treated with and without the HCQ. The weights were normalized with weight of the tissue. *, <0.05 compared with sham, **, <0.05 compared with AVF; *n* = 5–8 in each group.

Heart weight/body weight was increased in AVF mice hearts suggesting cardiac hypertrophy in AVF as compared to control mice. The treatment with HCQ mitigated this cardiac hypertrophy. Similarly, the lung weight/body weight was also increased in AVF mice indicating congestive (cardio-pulmonary) dysfunction in AVF mice; however, the treatment with HCQ attenuated this congestive heart failure phenotype (Figure 1).

### 2.2. Assessment of CK Isoforms, MMPs, and TIMPs Activities, and Myocytes Contractions

**Figure 2:**
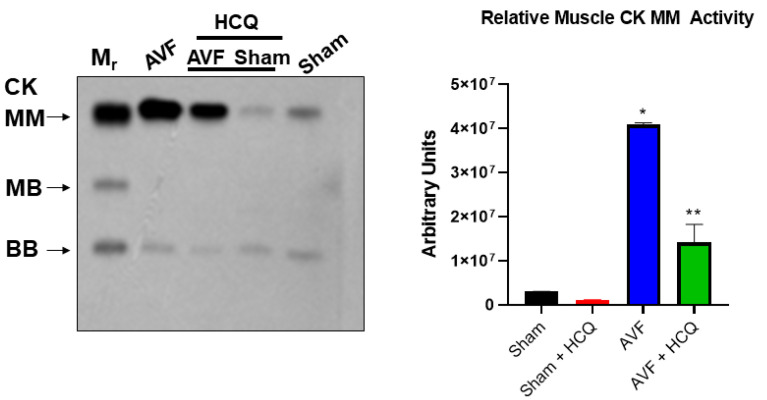
Representative serum in gel-muscle CK activity (left side gel picture); the bar graph represents the average scanned values of AVF and sham mice with and without the HCQ treatment (right side bar graph). *, <0.05 compared with sham, **, <0.05 compared with AVF; *n* = 5–8 in each group.

**Figure 3:**
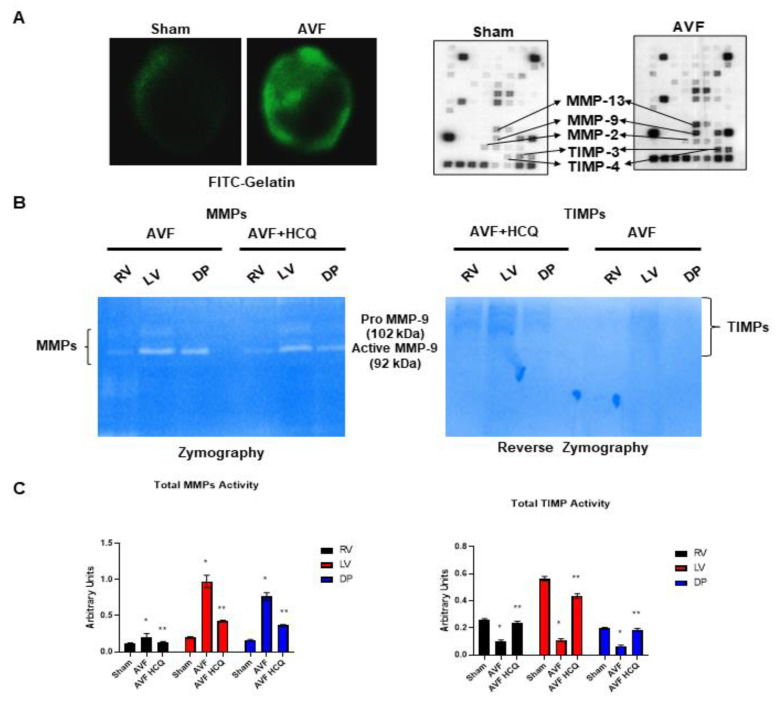
(**A**) Representative in situ FITC-Gelatinase activity and protein-array for MMPs and TIMPs levels in sham and AVF groups. (**B**) Representative MMPs gelatin-gel zymography and TIMPs reverse zymography from right ventricle (RV), left ventricle (LV) and diaphragm from AVF and sham mice treated with and without HCQ. (**C**) Bar graphs represent the average scanned values of AVF and sham mice with and without the HCQ treatment. *, <0.05 compared with sham, **, <0.05 compared with AVF; *n* = 5–8 in each group.

**Figure 4:**
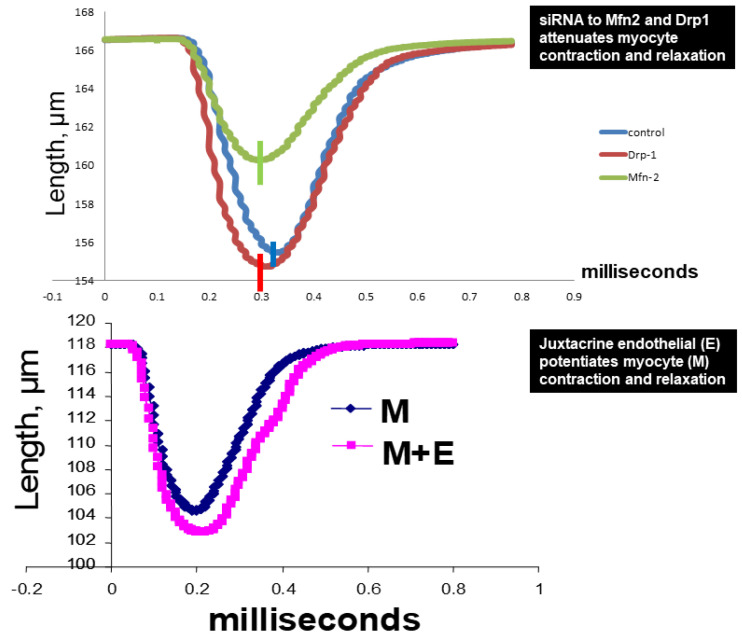
Ex vivo myocyte (M) contraction in the presence of siRNA to Mfn2 and Drp1, and endothelial cells (E). The contraction was measured in an ion-optic apparatus. *n* = 50–60 myocytes were used in each experiment.

The serum levels of in gel-muscle injury markers were robust in AVF mice but the treatment with HCQ slowed the muscular (skeletal and cardiac) injury in chronic volume overload-induced heart failure (Figure 2). Tissue remodeling implies the synthesis and degradation of ECM since MMPs and TIMPs play significant role in the cardiac remodeling. The results suggests that MMPs were robust in AVF as compared to sham whereas TIMPs were decreased. When the mice were treated with HCQ, the treatment normalized the levels of MMPs and TIMPs in CHF (Figure 3). Mitochondrial Mfn2 is a fusion protein while Drp1 is a fission protein. Therefore, these proteins play significant role in cardiomyocyte mitochondria fusion and fission and bioenergetics. Here, we measured single myocyte contraction in the presence and absence of siRNA of Mfn2 and Drp1. The results suggested that in the presence of siRNA, the myocyte contractions were attenuated (Figure 4). Additionally, because cardiac endothelial is juxtacrine to myocyte, we measured cardiomyocyte contractions in presence and absence of endothelial cells. Herein, the results suggested that endothelial controls myocyte contractions (Figure 4). These turned out to be the novel experiments.

### 2.3. Measurement of Contraction, and Relaxation of Ventricle Rings, and Microvascular Permeability

**Figure 5:**
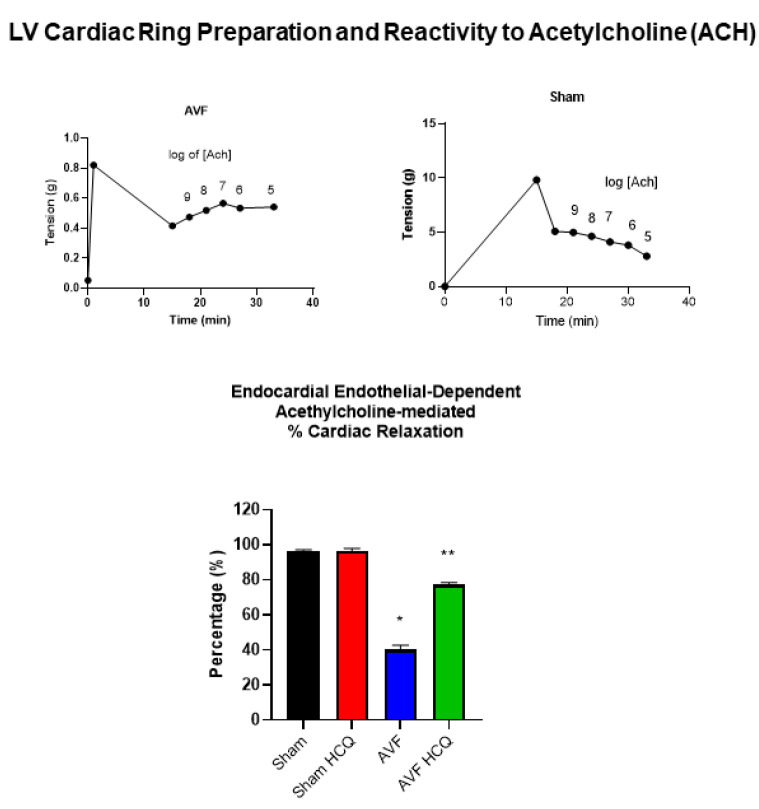
Left ventricle cardiac ring ex vivo preparation and endothelial-myocyte contraction measurement in myobath. Representative tracing from Sham and AVF (upper panels). Bar graphs (lower): % relaxation to acetylcholine (ACH), the rings were contracted with 20 mM CaCl_2_ and ACH was added. The % relaxation was estimated to 100% CaCl_2_ contraction from AVF and sham mice with and without the HCQ treatment. *, <0.05 compared with sham, **, <0.05 compared with AVF; *n* = 5–8 in each group.

**Figure 6:**
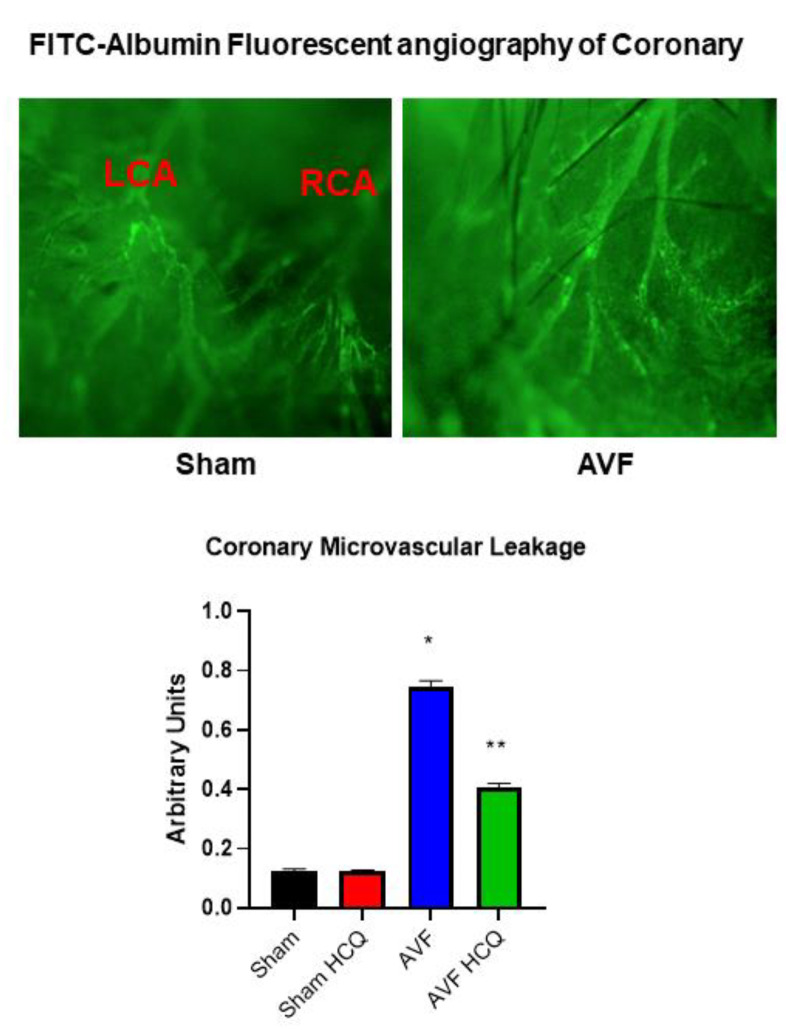
In vivo coronary microvascular permeability using FITIC-albumin: the upper panels are representative florescence angiography from sham and AVF hearts. The bars graph (arbitrary unit) displays AVF and sham mice with and without the HCQ treatment. *, <0.05 compared with sham, **, <0.05 compared with AVF; *n* = 5–8 in each group.

Because echocardiography presents whole heart function, we decided to separate LV from RV and from these ventricles we prepared the cardiac LV rings that were separate from RV and the atrium. By doing so, we were able measure the role of cardiac endothelial cells in myocyte contraction from the cardiac LV ring preparations. The results suggested that endothelial was impaired in AVF hearts and the same was mitigated by the treatment with HCQ (Figure 5). In vivo FITC-BSA was injected via the tail vein and the cardiac vascular fluorescence versus extravascular fluorescence were measured. The results demonstrated that there was robust leakage of coronary in AVF hearts in comparison to the sham hearts. Interestingly, the treatment with HCQ mitigated this leakage (Figure 6).

### 2.4. X-Ray Angiography, Ex Vivo Coronary Vascular Leakage, and Echocardiography

**Figure 7:**
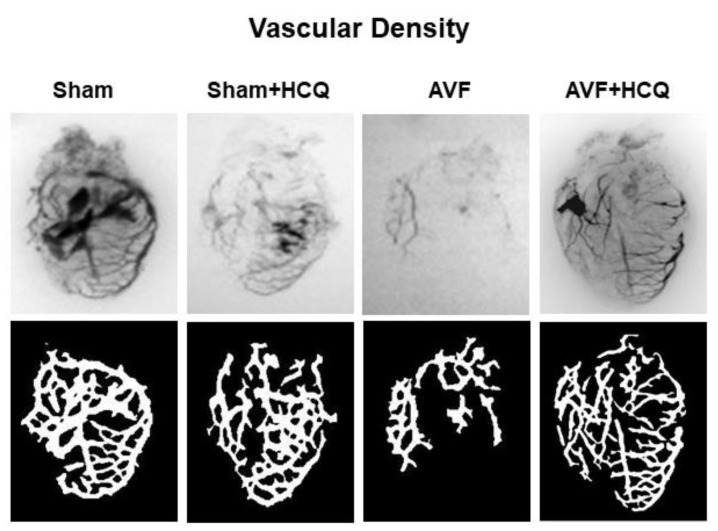
Representative barium X-ray angiography and vascular density from the hearts from sham, and AVF treated with and without HCQ: *n* = 5–8 in each group.

**Figure 8:**
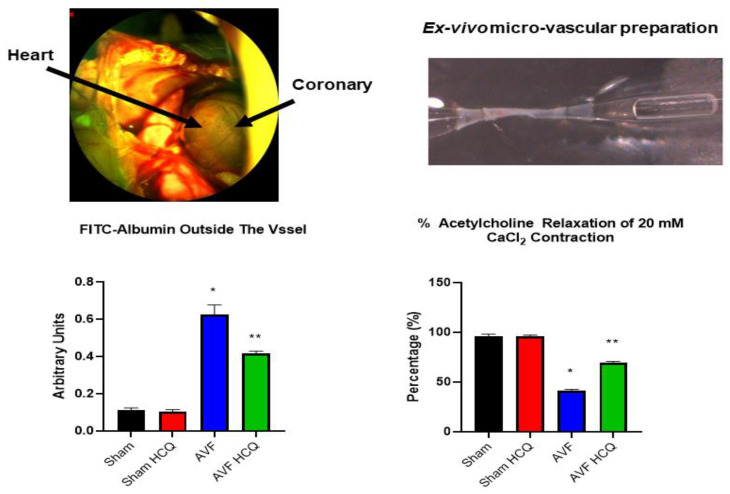
Ex vivo coronary vascular preparation and measurements of the vascular leakage and reactivity. The left upper panel shows a whole heart perfused with FITC; the right panel shows an isolated cannulated perfused vessel. The lower left bar graph represents FITIC florescence outside the perfused vessel. The right bar graph represents endothelial-dependent acetylcholine response to the coronary vessel, from AVF and sham mice with and without the HCQ treatment. *, <0.05 compared with sham, **, <0.05 compared with AVF; *n* = 5–8 in each group.

**Figure 9:**
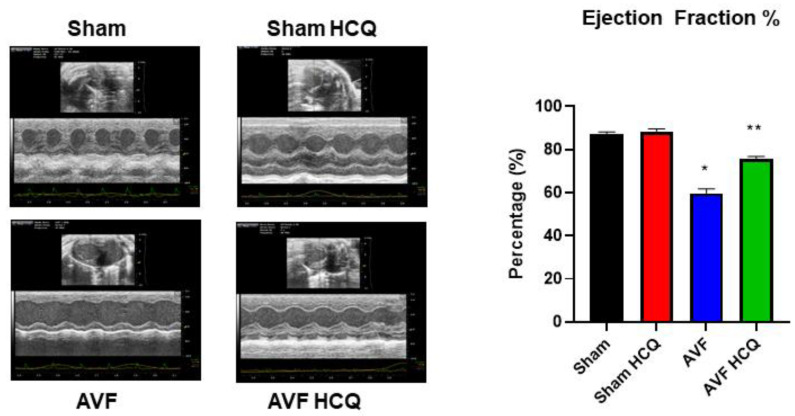
Representative scans of echocardiography; the bar graph represents % ejection fractions (EFs) of hearts from AVF and sham mice with and without the HCQ treatment. *, <0.05 compared with sham, **, <0.05 compared with AVF; *n* = 5–8 in each group.

The total vascular density in the heart was measured by barium sulfate contrast X-ray fluorography. The results revealed that there was a decrease in vascular density in the AVF hearts of the mice but treatment with HCQ seemed to normalize the vascular density post-AVF (Figure 7). Further, we also did the ex vivo vascular permeability measurement by knowing the leaking of FITC fluorescence in bath from inside the vessel perfusate, and as shown in the results suggesting that extra vascular fluorescence was, in fact, higher in AVF heart vessels. Again, the HCQ treatment normalized this very leakage (Figure 8). The coronary vascular endothelial dependent vascular relaxation was also measured in the cannulated vessel, and the results revealed a decrease in endothelial-dependent coronary micro-vascular relaxation; however, the treatment with HCQ reversed this decrease in endothelial-dependent coronary dysfunction (Figure 8). Then at the end, whole heart cardiac function was measured by performing the echocardiography. The findings suggested that EF was attenuated during AVF, but interestingly, the treatment with HCQ mitigated this attenuation of cardiac ejection function in CHF (Figure 9).

## 3. Discussion

Heart lung weights were increased in AVF-CHF, suggesting congestive heart failure post AVF in mice. The treatment with HCQ mitigated this increase in weights. Since AVF creates heart, lung, and vascular overload, we measured multiorgan damage by measuring the CKs activities. The findings suggested a robust increase in CK MM muscle activity indicating the multiorgan damage by chronic volume overload. Interestingly, the HCQ mitigated the multiorgan damage post treatment. Previously, we have shown that MMP-9 mediated the leakage of blood brain barrier (BBB) (24, 25), therefore, in this study MMP-9 mediated breakdown of BHB was measured. It is known that MMP-9 degrades EE connections and make EE leaky. Since CHF and AVF involve RV, lung and then LV dysfunction, it is thus important to measure the RV, LV and pulmonary diaphragm function and MMP-related muscle remodeling.

Several drugs are being tried for mitigating the COVID-19 symptoms including dapagliflozin (SGLT2-antidiabetic) lopinavir/ritonavir, darunavir/umifenovir (anti-HIV), remdesivir (anti-Ebola), favipiravir and dipyridamole (anti-hypertensive). We consider HCQ as a unique one in the sense that it mitigates unhygienic conditions related viral induced illnesses in the developing countries. Although we employed HCQ to test its efficacy whether it could help mitigate CHF in the mouse model, our work has relevance to the heart failure (HF) phenotype that is also the sequel of many viral myocarditis including the COVID-19 infections affecting the cardiovascular function in humans. Therefore, in future it would be important to further determine, in detail, whether the MMP-9 activation induces EMMPRIN (CD147), decreases connexin-37 and causes endocardial endothelial leakage and that HCQ mitigates MMP-9 activation, EE leakage and pulmonary diaphragm muscle dysfunction.

Although, MMP9 degrades collagen/elastin and connexins, the turnover of collagen is rapid as compared to elastin or connexins, that in turn, is replaced by stiffer collagen [80]. In this regard, a change in collagen/elastin ratio could lead to fibrosis. Higher cardiovascular risk in CHF, in fact, is attributed to fibrosis and MMP-9 that degrades connexins (−37 and −43), causes E-M, and M-M uncoupling. The fibrosis due to degradation of collagen by MMP-9 can be mitigated by down regulating the levels of MMP-9. Although HCQ may down regulate MMP-9, the mechanism is not known. Therefore, it is imperative to determine whether the MMP-9 activation decreases connexin-43 and causes endothelial-myocyte (E-M) and myocyte-myocyte (M-M) uncoupling and HCQ mitigates them. In myocytes, connexins are important for proper functioning of the mitochondria [81,82]. Elevated mitochondrial MMP-9 levels degrade connexin-43 that contributes to mitochondrial-myocyte uncoupling. We have shown that there is dysregulation of mitochondrial fusion and fission during heart failure [83,84]. The dynamic process of myocyte contraction and relaxation is synchronized with mitochondrial fusion and fission since mitochondria contribute significantly to the calcium balance and energy production in the myocytes [21]. There is a decrease in Mfn2/Drp1 ratio (Mfn2; mitofusion 2, mitochondrial fusion protein/Drp1; dynamin related protein, and mitochondrial fission protein) which indicates an abnormal fission over fusion prompting abnormal mitophagy [84]. The altered mitochondrial dynamics due to decreased Mfn2/Drp1 ratio leads to dyssynchronization. HCQ could mitigate mitochondrial-myocyte uncoupling by maintaining the mitochondrial dynamics (Mfn2/Drp1 ratio) and inhibiting MMP-9 mediated connexin degradation. Together our study suggests that HCQ alleviates the induction of the proteolytic and mitochondrial oxidative stress in the mice hearts [76,77,78,79].

## 4. Materials and Methods

### 4.1. Animal Protocol, and Treatment

Because aging and viral myocarditis induce CHF primarily due to chronic increase in preload, therefore, we created AVF below the kidney in adult 12–15 weeks age old male wild type (WT, C57BL/6J) mice. The animals were allowed to recover from the anesthesia, and then starting next day they were treated with or without HCQ (Hydroxychloroquine Sulfate, that was diluted in sterile water (0.005 g + 1.0 mL H_2_O) as an intra-peritoneal (IP) injection @100 µL/mouse, twice a week for a total period of 6 weeks. Control/sham mice were given only the sterile water. AVF was created in anesthetized mice between the aorta and caudal vena cava, ~0.5 cm below the kidneys, using a 30-gauge needle. A minimum of 5–8 mice were used in each group. Most of the animals survived the surgical procedure. On completion of the experiments, heart, and lung weights of AVF and sham mice treated with and without the HCQ were recorded and the weights were normalized with weight of the tissue. An infrarenal AVF creates an unambiguous model of chronic volume overload. In this model, arterial blood below the kidneys rapidly enters venous circulation and overloads the ventricles without contribution from the stimulation of circulating factors [85]. This model omits the effects of mechanical injury and vasodilatation [86,87]. We selected the C57BL/6J strain, as this mouse strain serves as the accepted background for genetically engineering the mouse species. Moreover, results by others have suggested extensive genetic homology between a mouse and human [88]. Because CHF due to AVF causes right ventricle (RV), pulmonary and left ventricle (LV) dysfunction, we will analyze RV, LV, and the pulmonary diaphragm. It also leads to oxidative stress and proteolytic activation concurrently. Therefore, to avoid early activation of MMP and to prevent AVF-mediated oxidative and proteolytic stress, HCQ (100 nM) is administered via IP before surgery and then twice a week as described earlier.

### 4.2. Plasma Creatine Phosphokinase (CK) Activity

Multi-tissue specific injury was determined by measuring creatine phosphokinase (CK) isoforms in the plasma as described earlier [89]. Briefly, isoform CK BB is primarily nerve and kidney specific, MB is cardiac, and MM is the cardiac and skeletal muscle specific. Here, serum samples were mixed with 5 µL of β-mercaptoethanol (BME) and loaded onto the 1% agarose gels. The gels were pre-equilibrated with CK substrate, and the catalysts as recommended by Sigma. The gels were run at 175 V for 45 min. The standard (ST) amounts of CK isoforms were loaded parallel to the samples. Based on the densitometric intensity (I) of the samples, the amount of specific CK isoform was estimated as follows: Amount_ST_/I_ST_ × I_Sample_. A modified version of CK assay is available wherein either plasma or serum samples could be mixed with 1 µL of activator and loaded onto the pre-made CK gel as instructed by the manufacturer (QuickGel^®^ CK Vis isoenzyme procedure; Helena Laboratories, Beaumont, TX, USA). The gels are typically run at 400 V for 4:15 min. The standard (ST) amounts of CK isoforms (that are supplied by the kit manufacturer as loading controls) were also loaded in parallel to the samples for comparison [90,91].

### 4.3. Zymography and Reverse Zymography

The in situ FITC-gelatinase activity and protein-array for MMPs and TIMPs levels in sham and AVF groups along with the matrix metalloproteinases (MMPs) and tissue inhibitor of metalloproteinases (TIMPs) activities were determined in whole heart and the tissue samples respectively prepared from the ventricle (RV), left ventricle (LV) and diaphragm. These tissues were collected from AVF and sham mice that were treated with and without HCQ and loaded in the gels and visualized as reported previously [92]. Type I gelatin was added to standard Laemmli acrylamide polymerization mixture at final concentration of 0.5 mg/mL [93]. Electrophoresis was then carried out under non- reducing conditions. Matrix metalloproteinase inhibitor activity was visualized by electrophoresis on SDS-substrate gels as described above. After the Triton X-100 step in zymography, gels were incubated at 37 °C in the presence of trypsin activated tissue extract for 1–2 h. After activation, in the extract 1 mM PMSF was added to inhibit trypsin. This tissue extract contains several proteinases that partially degrade the substrate within the gel under these conditions, except where inhibitors were located. Divalent metal ion chelators, such as 1,10-phen-anthroline, abolished the ability of activated extract to degrade substrate, confirming that this activity was due to metalloproteinase. The gels impregnated with activated cardiac extract metalloproteinases were then incubated overnight in substrate buffer, stained, and de-stained.

### 4.4. Myocyte Contraction

The single myocytes were isolated by collagenase digestion and contraction of single myocyte was measured using a modified protocol via the by IonOptix (Boston, MA, USA) as previously described [90,94,95,96,97]. In brief, the hearts of mice were dissected out after anesthesia and then perfused with freshly made perfusion buffer with Liberase TH [84,98]. The yield was about 80% and did not vary much between different group of animals. Isolated myocytes were used immediately for studying the contractility measurements. Ex vivo myocyte (M) contraction in the presence of siRNAs for Mfn2 and Drp1, and endothelial cells (E) was measured in an ion-optic apparatus. Additionally, the systolic and diastolic functions on isolated myocytes were also recorded. The decay of calcium transient was recorded as described [99]. A subset of cardiomyocytes was incubated with Fura-2-AM (1.0 µmol/L) for 30 min and the fluorescence measurements were recorded with a dual-excitation fluorescence photomultiplier system as described before after 1 Hz field stimulation [84]. Myocytes were field stimulated (at a frequency of 1.0 Hz, pulse duration of 4 ms and amplitude of 10 volts) using IonOptix myopacer and the contractions were recorded through SoftEdge^TM^ Acquisition Software version 9 [84]. Randomly selected, five myocytes were recorded for contraction parameters at a time from an unstimulated pool and a total of 50 to 60 myocyte recordings per heart were collected for the analysis.

### 4.5. Cardiac Ring Preparation, and Endothelial Myocyte Coupling

The determination of endothelial function in an isolated papillary muscle preparation does not demonstrate what happens in the entire trans-myocardial wall [100]. Therefore, to determine endocardial endothelial (EE) function, acetylcholine was perfused in a Langendorff preparation [101]. However, this does not differentiate the specific contribution of the regional ischemia, hypertrophy, stunning, and/or hibernation of myocytes in the myocardial wall. Rather it gives a global contractile response to cardiotonic agents. Furthermore, it does not separate the effects of the LV from the RV. Using cardiac ring preparation, we compared data obtained from cardiac rings with data obtained by Langendorff preparation in hypertensive rats and found similar results (49). In addition, the cardiac ring preparation separates the effect of LV from RV. To determine the specific regional differences in contractile function, the rings will be prepared to include or to exclude the homogenous or non-homogeneous regions of the transmural myocardial wall [101,102,103]. The endothelial dependent cardiomyocyte function will be measured in cardiac rings prepared from left ventricle and right ventricle (LV and RV) separately from intact and endothelial-denuded hearts from sham and AVF mice. The response to acetylcholine (an endothelial-dependent) and nitroprusside (endothelial-independent) relaxation will be measured.

### 4.6. FITC-Albumin Perfusion, and Vascular Leakage

The endothelial permeability was measured in the coronary that were perfused with FITC. The measurement of florescence outside the coronary was recorded [104]. Similarly, the endocardium was also perfused with FITC-albumin and fluorescence was analyzed by the fluorescence microscopy.

### 4.7. Barium Sulfate Contrast X-ray Angiography

To determine vascular density barium sulfate contrast, X-ray angiography was performed as per the method described in our previous work [105]. In short, the barium sulphate has been widely used by the radiologists in “Barium meal”, “Barium swallow” and “Barium enema” preparations to visualize the structural and motility abnormalities of the gastrointestinal tract mostly in the pediatric population. Though iodinated contrast agents are mostly used for intravascular imaging, with small animals such as mice and basic X-ray, we found that the barium gives better vascular imaging than iodine compounds since the size of barium particles ranges from 1 to 100 µm, depending on the solubility of barium sulphate in aqueous solution [106]. We dissolved barium sulphate in 50 mM Tris-buffer (pH 5.0) and infused slowly at a constant pressure and flow with a syringe pump at 200 µL/min using the intravascular route; this produced the optimal visualization of vascular density. Animals were dissected open to expose various organs, and angiograms were performed. All images were taken with Kodak 4000 MM image station. Dissected animals were placed in the X-ray chamber and angiograms were captured with high penetrative phosphorous screen by 31 KVP X-ray exposures for 3 min and aperture settings of approximately 4.0, f-stop-12 and variable zoom for different organs.

### 4.8. Coronary Leakage Due to Endothelial Dysfunction

A mixture of FITC (300 µg/mL) and BSA-647 (3.3 mg/mL) in PBS ~100 µL solution was infused through the tail vein injection in the anesthetized mice and FITC-BSA and allowed to circulate for 30–40 min. The in-situ leakage of the coronary was observed by exposing the hearts after performing thoracotomy under the microscope. The microscopic images were acquired. The results were averaged for each experimental group as per the protocol described [107,108,109].

### 4.9. Echocardiography, and Cardiac Function

To determine overall myocardial function, Echo was performed as previously described [90]. Briefly, an ultrasound was performed using the Vevo 2100 imaging system. The cardiac and aortic data were collected as reported earlier [110]. The mice were placed supine on a warm platform having a temperature of 37 °C under the effect of isoflurane anesthesia and fixed. Then using a MS550D (22–25 MHz) transducer, thoracic cavity was imaged, and aortic arch velocity and cardiographic functions were recorded in pulse wave and color Doppler modes. Transducer probe was placed on the left hemithorax of the animals on the partial left decubitus position. Two-dimensionally targeted M-mode echocardiograms were obtained from a short-axis view of the left ventricle at or just below the tip of the mitral-valve leaflet and were recorded. LV size and the thickness of LV wall were also measured. Only the M-mode ECHO with well-defined continuous interfaces of the septum and posterior wall were collected.

### 4.10. Statistical Analysis

Data from sham, AVF, sham + HCQ and AVF + HCQ were collected and statistically analyzed using the one-way ANOVA to analyze the difference between the groups, including a Tukey’s post hoc analysis for groups’ comparison, comparing AVF with sham and AVF + HCQ with AVF groups. Data are Mean ± SEM (*n* = 5–8 animals/group), * *p* < 0.05, ** *p* < 0.01.

### 4.11. Ethics Approval

The animal procedures were reviewed and subsequently approved by the Institutional Animal Care and Use Committee (IACUC) of the University of Louisville School of Medicine, Louisville, KY, USA. Further, the animal care and guidelines of the National Institutes of Health (NIH, USA) were also adhered to during all the experimental procedures.

## 5. Conclusions

The primary cause of CHF leading to death during old age and viral myocarditis is not fully understood. The goal of this project was to determine the mechanism of BHB during CHF. Using an animal model of CHF by creating AVF, the control as well as AVF mice were treated with HCQ. Both structural, as well as functional parameters were measured. Our findings reveal that metalloproteinase activation, EE leakage, E-M uncoupling, altered Mfn2/Drp1 ratios, and MM uncoupling during AVF heart failure were the abundant features but treatment with HCQ could successfully mitigate deleterious CHF phenotype highlighting the potential application of CHQ as a possible therapeutic option for the clinical consideration [76,77,78,79].

## Data Availability

All data and other materials are available upon request from the authors.
